# Aligning with the flow of control: A grounded theory study of choice and autonomy in decision-making practices of people with intellectual disabilities

**DOI:** 10.1080/17482631.2020.1857053

**Published:** 2020-12-17

**Authors:** Eileen Carey

**Affiliations:** Department of Nursing and Midwifery, Faculty of Education and Health Sciences, University of Limerick, Limerick, Ireland

**Keywords:** Choice, control, autonomy, self-determination, decision-making, intellectual disability, grounded theory

## Abstract

**Purpose**: Choice and autonomy are recognized as values facilitating genuine self-determination. Subsequently greater understanding of these concepts in decision-making practices of adults with intellectual disabilities is required.

**Aims**: The twofold aim of this research study was to ascertain the core concern (most important issue) for adults with intellectual disabilities as they make choices and exercise autonomy and to develop a theory explaining how these adults attempt to resolve their core concern.

**Methods**: This research study undertaken in a single organization in the Republic of Ireland applied classic-grounded theory methods. Participants included twelve adults who were attending day services and accessing a variety of other organizational services. Interviews were undertaken, between January 2012 and September 2013, in different contexts on up to 4 occasions (46 interviews). Data analysis utilized concurrent processes of constant comparative analysis.

**Results**: The main issue of concern for these participants was ‘control’ in environments that were controlling of them and they responded by ‘aligning with the flow of control’ explained by how they framed control, emotionally connected and adjusted in compliance situations.

**Conclusions**: This theory offers a conceptual delineation of the way adults with intellectual disabilities manage the daily tensions and harmonies in decision-making.

## Introduction

Individuals who ‘own’ their lives make choices that are inherently tied to a sense of self, which is reflected in the traditional philosophical concept of autonomy (Moore, 2019). Legislators for international human rights advance concepts of autonomy and independence by means of emphasizing the capacity of people for social, physical, emotional and cognitive development (United Nations Convention on the Rights of Persons with Disabilities [UNCRPD], 2006; Zolkefli, 2017). Thus, Article 3(a) of the United Nation’s Convention on the Rights of Persons with Disabilities obligates societies to uphold ‘ … *individual autonomy including the freedom to make one’s own choices and independence of persons;*” (UNCRPD, 2006, Article 3(a)). In a practical sense, autonomy is the ability of an individual to direct how he or she lives on a day-to-day basis according to personal values, beliefs and preferences (Health Information and Quality Authority [HIQA], 2019). And, the stronger personal autonomy is advanced and productive in societies, individual citizens are enabled to become best self-advisors on compliance to his/her expertise and life style (Gumbis et al., 2017).

Choice Theory is an internal control psychology that occurs as four distinct, but inseparable components of acting, thinking, feeling, and physiology happen simultaneously as individuals make choices which are driven by genetic needs of survival, belonging, power, freedom and fun (Glasser, 1998). Choice theory stipulates that each individual possesses a mental set of pictures based on past experiences and future aspirations and aligned with current needs of what s/he considers to be their ‘quality world’. Understanding and supporting the individuals ‘quality world’ will enable the person find satisfaction in their relationships with- relevant others so engaging in more internal control psychology (Glasser, 1998).

Historically, mainly due to limiting societal and cultural responses to supporting individuals with intellectual disabilities, this population group found themselves segregated from their societies, institutionalized and consequentially lacking autonomous opportunities to live similar lives to the general population (Brown & Brown, 2009). Due to the knock-on effect of living segregated lives in mainly group-managed services, inevitably, these individuals have been subjected to restrictions of choice in their lives. They experienced limited opportunities to engage and participate not only in making decisions for themselves as individuals but also in contributing to group and wider society decisions (Carey & Ryan, 2019). Whereas today, international and national policies and legislations are devoted to enabling conditions so that people exercise the right of choice (Carey & Griffiths, 2016; UNCRPD, 2006). Having choice opportunities is a precursor to potentially empowering autonomous outcomes for people with intellectual disabilities which must be understood if robust responses are to be developed to implement the principles of the UN Convention (UNCRPD, 2006).

Brown and Brown (2009) identified a five-step strategy for integrating choice into the lives of these individuals: (1) assessment of choice acceptance in the environment; (2) clarification of methods for broadening familiar choice opportunities; (3) clarification of methods to increase individual’s freedom, initiative, and skills in choice-making; (4) increase skills of relevant support persons; (5) development and assessment of specific methods for recording choice in practice which link choice with other key quality of life concepts. Webber and Cobigo (2014) advocated that the following components should be used as measurements to evaluate services for people with intellectual and developmental disabilities: (1) the availability of choice opportunities; (2) the provision of choice options; (3) informed cognitive processes and act of choosing, and (4) a supportive environment. Webber and Cobigo (2014) found methods to promote choice to be limited and recommended further research be undertaken to assist service providers evaluate their services, demonstrate accountability, and to maintain a balance between promoting choice and protecting vulnerable adults. Policy, practice and academia discourse within the field of intellectual disability, continues to feature choice definitions in the context of presence or absence of related attributes reflected in the range of available measurements (Agran et al., 2010; Hatton et al., 2004; Houseworth et al., 2018; O’Donovan et al., 2017)

Research evidence while gaining prominence on choice processes and supports for people with intellectual disability, is in it’s infancy with regard to the actual choice experiences of adults with intellectual disabilities. Choice processes are evidenced as choice making exercises underpinned by: being motivated (Agran et al., 2010; Lysaght et al., 2009); identifying and selecting preferences (Donelly et al., 2010; McCarthy, 2010; Wiltz & Kalnins, 2008) and goal setting and problem-solving (Agran et al., 2010; McConkey & Collins, 2010). Choice making supports are found to be having: opportunities to make choices (Jensen et al., 2012; McDaniels, 2016; Stancliffe et al., 2011) and a supportive network with good support (Chapman et al., 2011; Conder et al., 2010; McCarthy, 2010; McConkey & Collins, 2010; Michell, 2012). Other important resources to support choice making are acknowledged as: education and skill development (Chou et al., 2011; Lysaght et al., 2009; Stancliffe et al., 2011). More recent influential approaches such as personalization (Williams & Porter, 2017), and interactionist strategies (Cudré‐Mauroux et al., 2019) are recognized as influencing choice processes and supports.

Regrettably, choices of some people with intellectual disabilities have been unacknowledged and devalued (Johnson & Bagatell, 2020). Challenges to making choices have been recognized as: limited choice availability and opportunity (Ciulla et al., 2011; McCarthy, 2010; Stancliffe et al., 2011); limited resources, for example, accessible information and meaningful consultation (Conder et al., 2010; Chou et al., 2011; Kilcommons et al., 2012; McCarthy, 2010; Michell, 2012; Owen et al., 2008). Other resources such as transport and financial disincentives were also cited as challenges to enabling adult choice to participate in meaningful activities (Lysaght et al., 2009). And attitudes of relevant others impact on choices made (Antaki et al., 2009; Benedick and Dixon 2009; Michell, 2012; Owen et al., 2008).

Similarly, research evidences the rise of differing angles on matters of autonomy for people with intellectual disability as reflected in: actualizing autonomy in daily lives (Bjornsdottir et al., 2015); implementing self-directed support (Bogenschutz et al., 2019); examining voting rights (Redley et al., 2012); health self-advocacy training programmes (Feldman et al., 2012) and autonomy support (Alonso-Sardón et al 2019; Frielink et al., 2017, 2018; Hawkins et al., 2011). As society seeks to operationalize such legislations and policies in practice discourse on matters such as processes and experiences of decision-making are brought to the fore (Bjornsdottir et al., 2015; Chou & Lu, 2011; Jenkinson et al., 1992; Jenkinson & Nelms, 1994; Rogers et al., 2020; Suto et al., 2005; Whitehead et al., 2016), supporting decision-making (Bigby et al., 2019; Carey & Ryan, 2019; Curryer et al., 2019; Gore, 2008; Shogren et al., 2017; Watson, 2016) and associated policy and research (Flynn & Arstein-Kerslake, 2014a, 2014b; Bach & Kerzner, 2010; Carney, 2013; Jenkinson, 1993; Kirkendall et al., 2017).

Ireland’s commitment to a national reform of disability service provision and a broader commitment to a right to live a life of one’s choosing either in one’s own home or in the community, while accessing services which are genuinely person-centred, has been reflected in the publication of key government policy documents and objectives (Health Service Executive [HSE], 2018, Irish Human Rights Equality Commission [IHREC], 2019; HIQA, 2019). It is anticipated that the recently ratified Assisted Decision-Making (Capacity) Act (Department of Justice, 2015) provides a legal framework for supporting decision making for people who require such support, and will result in significant improvements in the lives of persons with intellectual disabilities as their ability to make decisions for themselves will be enshrined in law. In doing so, it demonstrates a seismic cultural shift away from a paternalistic and ‘best interests’ approach towards persons with intellectual disabilities to a rights-based approach to choice and autonomy in decision-making. However, strengthening the commitment to human rights as endorsed in theoretical, legal and practice documentation has not been sufficient enough to guarantee and protect individuals with intellectual disability, as for many, the universality of human rights has not yet been fully realized (Carey & Ryan, 2019).

Researchers have indeed approached the issue of decision-making among adults with intellectual disabilities from various angles and using various methodologies; however, a comprehensive model of this process and its contributing factors has not yet been elucidated. To date there is a dearth of research representing the experiential realities of how adults with intellectual disabilities make choices and exercise autonomy. This article delineates a qualitative investigation into choice opportunities and the exercise of autonomy as reported by adults with intellectual disabilities in the Republic of Ireland who live in a society in which the government is actively implementing policies to promote greater choice opportunities, and the theory that emerged from that investigation. The twofold aim of the research study was to: a) ascertain the core concern (most important issue) for adults with intellectual disabilities as they make choices and exercise autonomy in their decision-making and b) develop a classic-grounded theory explaining how these adults who access support services attempt to resolve their core concern.

## Methods

**Figure 1.**
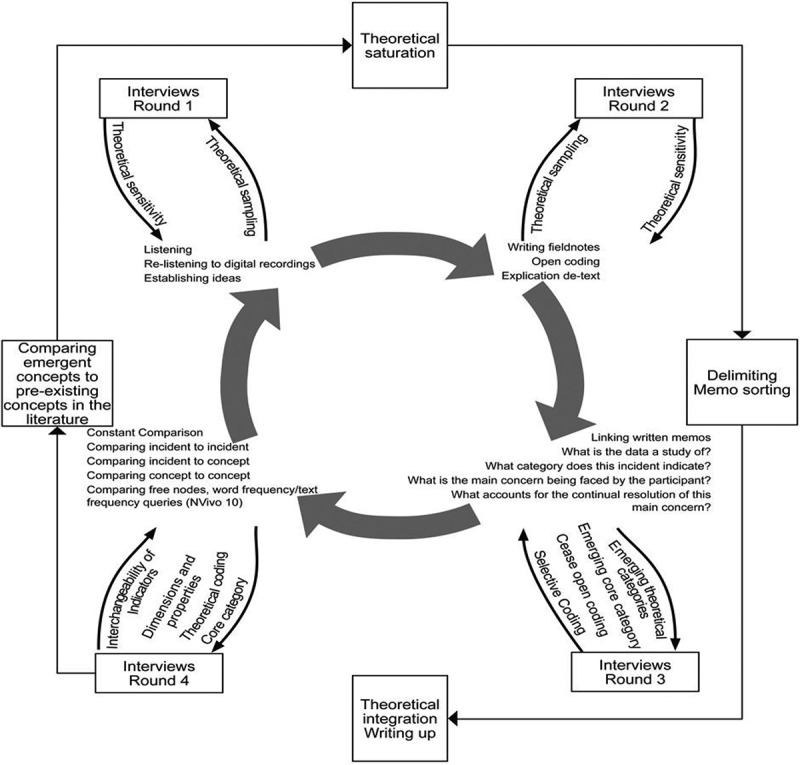
Overview of application of classic grounded theory

Figure 1 provides an overview of the application of classic-grounded theory methodology as applied in this research study:

### Design

Classic grounded theory is a systematic, inductive methodology founded on the premise that the problem emerges from and is guided by data collection, to develop a conceptual theory explaining a latent pattern of behaviour (Glaser, 1978, 1998, 2001). It relies on abstract conceptualizations and conceptual relationships while avoiding contextual descriptions and descriptive interpretations of the empirical data, whereas other qualitative research approaches focus on in-depth descriptions (Carey, 2010) or are overly reliant on literature to the detriment of developing a conceptual theory grounded in data from the substantive area. A classic-grounded theory building approach is more likely to generate new and accurate insights into the phenomenon under study than reliance on either past research or office-bound thought experiment (Glaser & Strauss, 1967). Classic grounded theory is a methodology most suitable for use in areas where scant research has been undertaken. Research which is inclusive of people with intellectual disabilities is still in it’s infancy. Classic grounded theory approach based within the context of lived experience, responsiveness to emergent themes and constant comparative method of data analysis was the chosen approach used to generate a conceptual explanation of choice and autonomy relevant to the decision-making of adults with intellectual disabilities.

### Participants

Participants were sourced within one organization from two different day-services day services, each of which, catered for the needs of approximately forty adults with intellectual disabilities.

The initial purposive sample had inclusion criteria of aged 18 to 65 years inclusive, currently in receipt of a community-based day service, who had experience of agreeing and disagreeing in the context of life choices and want to participate in the study. The service ethics committee required all adults opting to participate in the research study to be ‘deemed to be in generally good mental and physical health by an appropriate healthcare professional’. While the researcher had no control over the decision, this specific inclusion criterion had knock-on effects on the length of time it took to recruit participants delaying the commencement of the research study.

The researcher met with the Administrator, Day Service Manager and other informal gatekeepers to explain the study and gather information with regard to the communication needs of potential participants. Fourteen potential participants were invited to an initial information session. The researcher provided different options of Information Briefing Sheets (e.g., more text less pictures vs less text more pictures) along with verbal explanations of the study informed by best communication practices adopted by using websites such as www.changepeople.co.uk/andwww.bris.ac.uk/Depts/NorahFry/Plainfacts. From the outset potential participants were afforded the opportunity to have a support person for the duration of the project. The researcher was available each day for one week to answer any queries potential participants may have about the study. Of the twelve adults who provided written informed consent to be interviewed and to have the interview audio-recorded, five opted to have a support person present for the informed consent interview and seven opted not to have a support person. The ongoing process of consent was endorsed throughout the research study. The researcher in a timely manner reminded each participant of the upcoming process expectations while being vigilant for signs of assent or dissent. Issues of recruitment and informed consent are available in detail in a previous publication (Carey & Griffiths, 2017).

### Data collection and analysis

Participants in the study were twelve adult participants (eleven females and one male) ranging in age from 28 years to 47 years old. In classic-grounded theory specific identification of the number of people to be invited to participate in the research is challenging, as the theoretical sampling that is intrinsic to classic-grounded theory is unknown at the beginning. Glaser (1978) suggests that initial data should be gathered from the individuals who are the best informants in the area. Fortunately, a basic tenet of classic-grounded theory is that “all is data” (Glaser, 1998, p. 8). In alignment with this tenet and optimizing opportunities for data collection and theoretical sampling within this research study, interviews and informal discussions, were held in various locations, across many activities and at different times during the day. Opportunities for further theoretical sampling, be it access to further participants, activities or locations, if had been required were to be negotiated with the gatekeeper.

Up to four rounds of interviewing comprised forty-six semi-structured interviews in different contexts and settings. Seated interviews lasted from thirty minutes to one hour and thirty-five minutes. Times varied from morning to afternoon to evening. Interviews were held in quiet locations in either of two day-services, community settings e.g., coffee shops, community residential settings and an apartment. In making the interview as accessible as possible the researcher identified herself and sought to establish a good rapport with the interviewee using incidental conversation to start the interview. Ice-breaking questions were utilized such as: How is your day going? Tell me about yourself? In acclimatizing the adult participant to the interview situation the researcher together with the research participant would review the participant briefing sheet encouraging the participant to talk about an image of his/her choice and by discussing photos deemed important by the participant in the context of their life experiences. In keeping with a grounded theory approach at the outset the interview topic guide questions were kept general and open (Table I).

**Table I.** Interview topic guide questions

Questions	
What are your dreams/goals? What are you doing to make those goals happen? What is helping you to achieve those goals? Is there something that you are not doing that you would like to do? Tell me about it? What happens when you do not want to do something that you are asked to do?	How do you tell people what you want to do? What happened when you ….? What information did you get in relation to? What format was the information in? How was the information presented? What did you understand about the information as it relates to … ? () life choice? How did you agree or disagree to … … () life choice? What is your previous experience in relation to () life choice? How did you agree or disagree to … . () life choice? Tell me about your Individual Programme Planning meeting?

An example of a finishing question was “What would you do if you won the lottery?” And thanking the participants the researcher would inform each of the next proposed steps of the study ensuring that the participant was comfortable to continue.

The rich data content of interviews guided theoretical sampling and directed the process of data collection. Grounded theory interviews rely on the emergent data to stimulate and generate discussion on the topic as relevant and important to participant (Glaser, 1978, 1998). The researcher spent time with research participants chatting in day service, community residential houses or walking to and from coffee shops. Furthermore, when interviewing participants in their residential settings, many participants volunteered the sharing of photos reflecting aspects of their lives. What was deemed to be most important was to explore what was happening when these adults were agreeing and disagreeing to life choices. The researcher in listening and re-listening to the audio-recordings emerged herself, noting open codes typing field-notes and associated memos. The field-notes facilitated the incorporation of the researcher’s own explanations and reflections as to what was happening in the realities of participants’ choices. As initial codes began to emerge from adult stories of choice, they were grouped into similar categories which initially focused on outcomes. Classic grounded theory methodology involves a core process of coding, which comprises two types: substantive coding, which included both open and selective coding (Glaser, 1978). The constant comparative method enabled the researcher to look for patterns in the data and to compare emergent codes for similarities and differences (Glaser, 2001). Codes that frequently appeared during the open coding phase of initial interviews were related to participant reports of ‘being told what to do’. These initial codes were the foundation on which the analysis progressed and these formed the basis for the development of selective codes. Examining the data for interchangeability of indices and elaborating on memos the frequency with which the indicators were occurring enabled the researcher to make connections and to challenge researcher assumptions. Constant comparison of categories featuring outcomes and key choice experiences enabled identification of concepts to follow up for theoretical sampling and the collection of rich data explicating further connections in the data. Theoretical sampling for choice-associated concepts was undertaken, between and in participant sampling, occurring in different context and settings, with varying focus including living arrangements, money, food, shopping, friendships, relationships, travel, health access and service provision.

Follow-up interviews enabled clarification of issues previously raised and concepts projected by the adult participants as the main concern was identified. As substantive codes emerged the researcher integrated hypothesis testing into the interview process. This involved seeking clarification from participants about specific emerging hypothesis, for example, in relation to specific choice opportunities, relationships, public transport, medication management, the interviewer asked subsequent participants if they were ‘being told what to do’ or ‘doing it themselves’. Substantive categories then subsumed some open codes. Analysis then moved onto comparing and contrasting adult identities and differing mindsets and the interpretation of choices situations as possible predictors for control beliefs. Focusing on choice availability the researcher sought to identify if factors which influenced control beliefs were internally or externally negotiated and if and how indicators that control beliefs related to a persons’ emotions or adjustments in compliance situations. The researcher recorded key incidents and significant statements that related to emergent categories. As conceptualization evolved the data were interrogated for links between adults who were more self-directed in the choices they made and others who reported that they were continually being told what to do (externally directed). As concepts were emerging it was becoming obvious that a persons’ emotions, and the adjustments they made to comply were counterbalancing agents for determining the person’s control beliefs. Once the core category begun to emerge, the researcher selectively focused on the emergent categories explicating properties and dimensions. Incorporating the use of theoretical codes and comparing emergent concepts to pre-existing literature the researcher explained the relationships between categories. On reaching theoretical saturation, the researcher focused on memo sorting and delimiting to write up the theory. Once theoretical saturation of the core concept of framing control was achieved the researcher proceeded to review all data, field notes, memos and then sorted by category. Saturation occurred when up to four rounds of interviews had been conducted and no new themes identified.

### Ethical considerations

Ethical approval obtained from the University Faculty of Health Sciences Ethics Committee and from the Service Ethics Committee. Opportunities and challenges of navigating ethical approval while undertaking classic-grounded theory methodology are detailed in a previous publication (Carey, 2010). At the outset of each round of interviews, the researcher reminded research participants of the associated nature and procedures of the research study, allowing participant’s time to discuss with others and consider options. This ensured participants’ contentment in continuing with the research study. Participants were reassured of confidentiality when participating in the study and were informed in advance that in accordance with Ireland’s Code of Professional Conduct and Ethics for Nursing and Midwifery, it would be broken if the interviewer as a registered nurse Intellectual Disability (RNID) became concerned for the health and wellbeing of the research participant (Nursing and Midwifery Board of Ireland [NMBI], 2014).

### Findings and analysis

Twelve adults with intellectual disabilities participated in this research study. They ranged in age from 28 years to 47 years old and all attended day service provision. Participant demographic data is outlined in Table II.

**Table II.** Participant demographic data

No.	Name	Age	Gender	Married Single in Relationship	Living arrangementCommunity Residential Service (CRS)	Education Training	Employment	Voluntary work	Day-service	Family/Support Network	Mobile
P1	Saoirse	38	F	R	CRS	No	No	No	Yes	Full time CRS	Yes
P2	Sophie	40	F	S	CRS	Human Rights Seminar	Bar Work (Cleaning)	No	Yes	Visits mum on special occasions Close friend—a nun	Yes
P3	Katie	43	F	S	CRS	Human Rights Seminar	No	Yes Nursing Home	Yes	Goes home every weekend to parents	Yes
P4	Aoife	45	F	S	CRS	Human Rights Seminar	No	No	Yes	Limited family contact—home once a year	Yes
P5	Elaine	41	F	R	CRS	Leadership & Advocacy Course	Secretarial Work	No	Yes	Mum Rip Meets Dad monthly	Yes
P6	Maria	42	F	M	Rents Apartment	Leadership & Advocacy Course	Hotel (Cleaning)	No	Yes	Parents RIP Contact with uncle monthly	Yes
P7	Harry	28	M	S	CRS	No	No	No	Yes	Living with both parents	No
P8	Aislinn	37	F	S	CRS	Leadership & Advocacy Course	No	No	Yes	Estranged relationship with family	Yes
P9	Sarah	39	F	S	CRS	Leadership & Advocacy Course	No	No	Yes	Home at weekends twice monthly. Fostered	Yes
P10	Liz	39	F	S	Lives at home with mother	Leadership & Advocacy Course	Church (Cleaning)	No	Yes	Goes home each evening to mother & sister. Parents separated	Yes
P11	Aly	32	F	S	CRS	Human Rights Seminar	Swimming Pool (Cleaning)	No	Yes	Goes home monthly x2 & hols to Mother, sister and brother. Dad Rip	Yes
P12	Caoimhe	47	F	S	Lives at home with brother	Leadership & Advocacy Course	No	No	Yes	Goes home each evening to Brother	Yes

**Figure 2.**
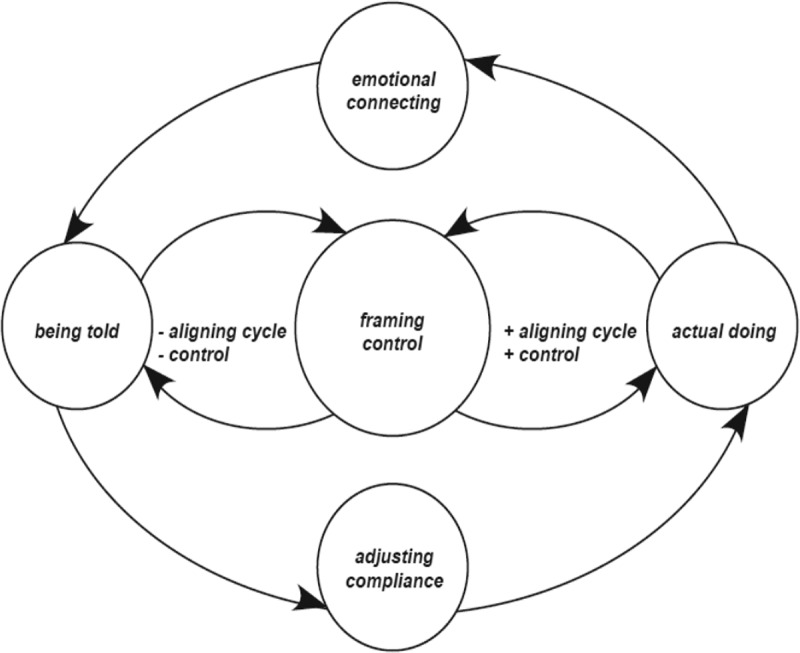
Diagrammatic representation of aligning with the flow of control cycle

**Figure 3.**
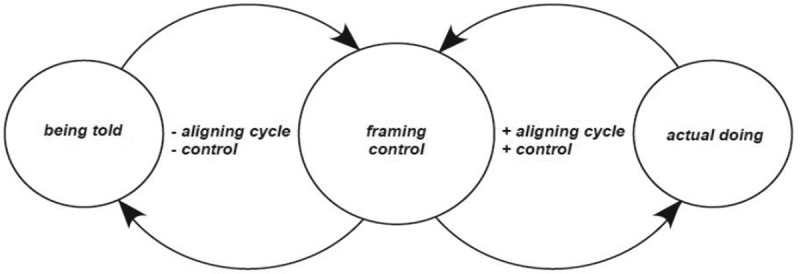
Diagrammatic representation of framing control

The following presents the findings of the theory of ‘aligning with the flow of control’. The main issue of concern for these adults is controlled in environments that are largely controlling of them. Having personal needs and aspirations, these adult participants live with intellectual disability, access group-managed services, engage with available supports, live with choice outcomes; all of which underpin their ‘actual doing’. Being told refers to external influences of government policy and legislation, social norms and enacting practices underpinned by agency (those in power positions/staff/family) attitudes and practices. These adults operate neither at one level (totally in control) nor the other (totally controlled), but move continually between these opposing spheres. For them, the conflict between ‘being told’ and their ‘actual doing’ determines and resolves by their ‘aligning’ patterns. And as they negotiate the multivariate and complex nature of life choices, this pattern of ‘aligning’ denotes equilibrium that exists for these adults, as they frame the control they have over the choice, counterbalanced by how they emotionally connect and adjust in compliance situations (Figure 2 and 3).

Framing control is a sub-core category of the aligning framework. It explains that drawing from a bank of ideas, adults make interpretations and position the control they have of the choice. The direction and extent of aligning is determined by the sub-core category of framing control, in which adults weigh up influencing contextual factors relating to ‘being told’ within the realities of their ‘actual doing’ and choose what to do next.

Frames of reference explain the bank of ideas which adults draw upon and project on to current choice arrangements. Many factors influence frames of reference, contextualized as identity frames, power frames, and mindset frames, which contribute to how adults frame control. Based on their past experiences and current realities, frames of reference are the grounding elements influential in guiding how they frame control. Factors such as values, customs and individual and group views are the foundations for ideas formed and stored as a bank of ideas to draw from when faced with the daily realities of what they are being told to do. They use these frames of reference as counterbalancing coordinates, leading to, and indicative of how they frame control. Frames of reference may be positive or negative, ingrained or open to change, and influenced by themselves and agency. The following presents an overview and excerpt on ‘identity frames’ a concept which contributes to ‘frames of reference’ which is a concept of the category-framing control. The following provides and samples of identify frames.

Identity frames denote the nature and extent of how adult experience being a human and being an adult. Although they have each attained the legal majority of adulthood, the social norms which set the behavioural expectations of adult citizens mean that these adults grapple with their identities on a fundamental level. The greater the deviation between what they are actually doing and social norms the more negatively they formulate frames of reference and vice versa. Positive identity frames represent affirmative experiences of being a human, such as ‘being valued’, and ‘having contributions recognized’, and of being an adult, in terms of ‘doing similar things to what adults do’, ‘having a job’, and ‘having a boyfriend’.

And examples of positive human identity frames:

Aly Yea … . Mammy, my mammy keeps missing me when I’m not at home. Yea, for making tea and coffee she misses me, yea, for taking the dog for a walk my mammy misses me … she misses me help … .my mammy misses me … for taking the dog for a walk

Katie I do voluntary work in XXX. At Christmas they asked me to the staff night out. We had a lovely meal. It was a lovely night.

An example of positive adult identity frames:

Caoimhe I phone the cab myself. I know the man. He collects me and brings me to the day service in the morning and in the evening I phone him and takes me home when I want.

Liz I have my own bank account and my own bank card

Negative identity frames explain reported experiences which lack humanity, such as: ‘being moved’, ‘being a number’ (e.g., when going shopping or on the bus), ‘not having a place to belong’ or being refused adult status, such as, ‘being treated like babies’, ‘being treated like a child’, ‘being ridiculed’ and some participants were critical of themselves. The contextualization of identity frames explains their perceptions of being human and adult. Examples of negative human identity frames:

Aoife I had to move to XXX house at Christmas. The houseparent just told me I had to move. I didn’t want to move to XXXX house. I don’t get on with the girls there. I told the houseparent I didn’t want to move and she told me I had to do it … .that’s what she told me … .she told me I had to move. she (houseparent) said—[‘that’s what I was told -so you have to go’]

Aislinn I got used to people putting me down. I got used to it. I find it hard to say nice things about myself. I do. I find it hard to say nice things about myself. I do find it hard to say nice things about myself, I do. I wish I was normal. It hurts me. I can’t understand I have a learning disability and my brothers and sisters haven’t. I can’t understand that and my nieces and nephews don’t have it how come I have it? When I see them I say why can’t I be normal? It does hurt me when I see them normal. It does hurt me. I hate it, having a learning disability. I do hate it, I hate it, I do hate it, I do cry about it. I have to live the rest of my life with it.

Aislinn I broke up with my boyfriend about a year ago. He wasn’t right for me, he was calling me names and everything, he was calling me ‘fatty’ and ‘four eyes’ and all this, I give [gave] him the boot, I give [gave] him the boot about a year ago.

Caoimhe I used to get the bus but there were boys who used to call me names and make fun of me.

And examples of negative adult identity frames:

Aislinn They (houseparents) treat us like children. I’m not allowed to make my own tea, pour milk or butter bread or make sandwiches for my lunch … . Treating us like babies.

Harry I’m just … .I’m not sure. It’s just … . it’s the cuts … . they are going to cut the transport. Well … … … .we think they are going to cut the transport. But we don’t know for sure. If they cut the transport … … . then … … … … then … … … . I don’t know how I’m going to get to the dayservice.

Saoirse The house parents set the house rules … they just tell us what to do …

Liz The xxx says “Liz you need to get your … .your … .check-up like, you need to go to the doctor, I will talk to your sister”. There’s no need to talk to my sister, like, I tell her there’s no need to talk to my sister and she says “no I will talk to your sister”

Whether positive or negative, the above examples of identity frames lead to framing control.

Adults continually draw from frames of reference based on the interactions between what they are being told to do and what they actually do. Whether agency enacting practices are more or less intensified, whether their own functional diversity is low or high, or whether optimal engagement exists, they draw on frames of reference to increase contextualization of framing control.

Choices made situate in the customized, multivariate and ever-changing conditions that affect them determine the nature and extent of aligning. For these adults, ‘being told’ is generally fixed, and even though negotiations are allowed, compliance is usually a requirement. Ultimately, regardless of consistency or changes in choice situations, it is how adults frame control that aligning cycles are set to move in either a positive (to the right) or negative (to the left) direction. The terms positive and negative inspired by the notion of homoeostasis, explains that framing control positively (higher levels) or negatively (lower levels) initiates changes or sustains stabilization in equilibrium for the person themselves.

Emotional connecting explains differing levels of emotional stability of adult participants. Lower level ranges of ‘de-stabilizing’ and ‘self-preserving’ and higher levels of ‘self-protecting’ and ‘stabilizing’. Adjusting compliance explains differing levels of participant adjustment in compliance situations ranging from lower levels of ‘dissentious’ and ‘selective’ to higher levels of ‘functional’ and ‘model’ complying.

The presentation of these cycles is intended as a framework for practitioners to understand the interactive nature of choice and control in decision-making. In normalizing routines, effecting or adapting to change, ‘aligning’ maintains, creates or changes the state of equilibrium that exists in interactions as the adult responds to matters of control. ‘Aligning with the flow of control’ conceptualized as a bi-directional positive or negative aligning cycles, denotes the equilibrium that exists in patterns of adult behaviours as they respond in the choices they make. Generally, at one extreme adults assimilate what they are told to do, creating personalization, ownership and are mostly self-directed in their decision-making, or at the other extreme, externally directed adults retreat back within the remit of expected roles of subordination.

**Figure 4.**
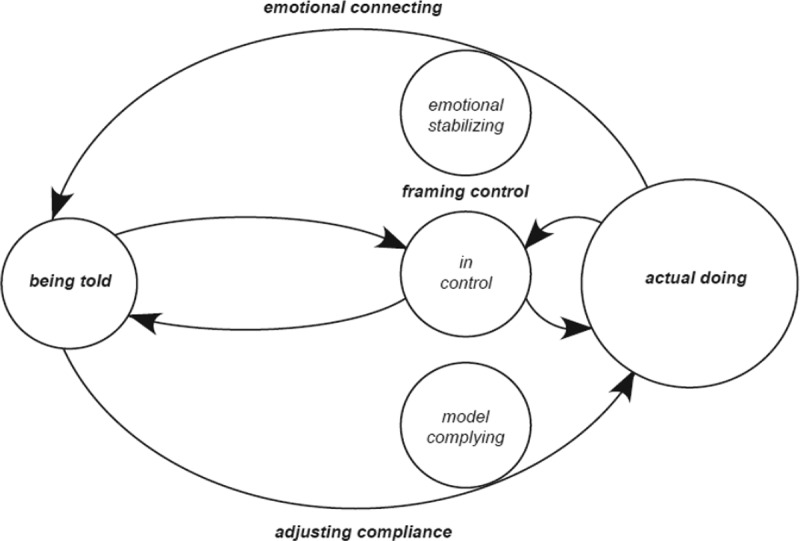
Diagrammatic representation of embracing the flow of control cycle

**Figure 5.**
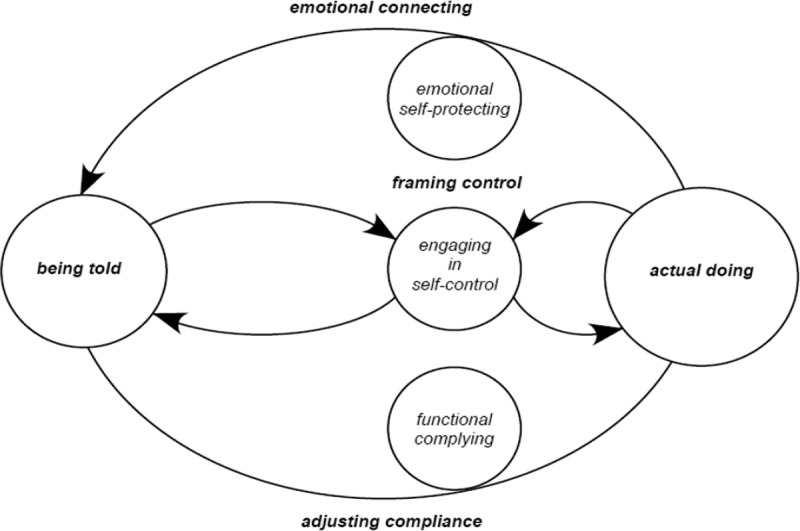
Diagrammatic representation of going with the flow of control cycle

For the purpose of clarity, let’s consider the interactive cycle in two halves, with positive aligning cycles oscillating to the right and negative cycles to the left. Positive aligning cycles underscore adults who are more self-directed rather than externally directed in their ‘actual doing’ (Figure 4: *embracing the flow of control* and Figure 5: *going with the flow*). Those with higher levels of control beliefs frame control more positively setting the direction of a positive aligning cycle moving the equilibrium towards amplifying their ‘actual doing’ rather than “being told’ *I make my own choices ”or ‘I just ring them and tell them where I’m going and what time I will be back’* Positive aligning cycles are indicative of and counterbalanced by adults with higher levels of emotional stability adjusting well in compliance situations. A positive aligning cycle minimizes the significance that compliance is a requirement of being told. The greater the beliefs of control the adult has the more motivated they become to continue behaviours that accommodate autonomy.

**Figure 6.**
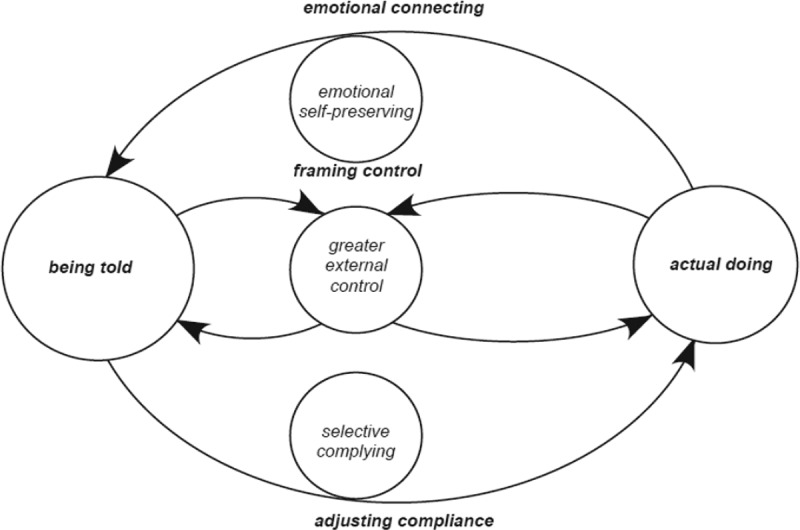
Diagrammatic representation of getting carried with the flow of control cycle

**Figure 7.**
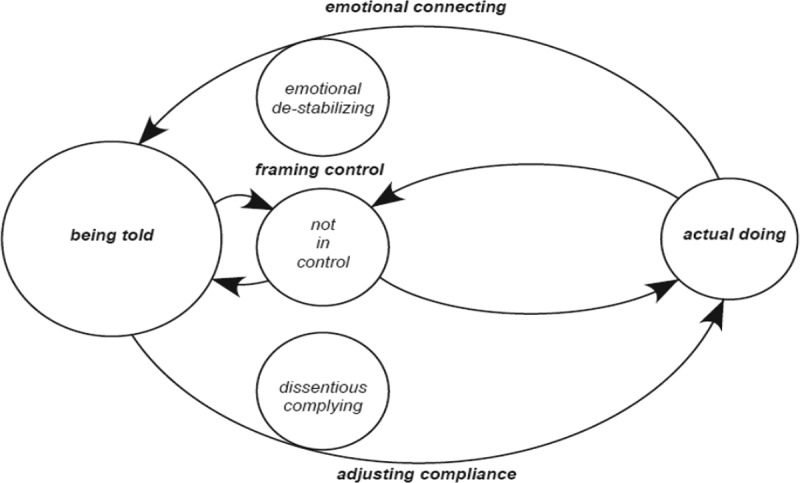
Diagrammatic representation of resisting the flow of control cycle

Negative aligning cycles underscore adults who are less self-directed and more externally directed in their ‘actual doing’ (Figure 6: *getting carried by the flow of control* and Figure 7: *resisting the flow of control*). Those with lower levels of control beliefs frame control more negatively setting the direction of a negative aligning cycle moving the equilibrium towards amplifying they are ‘being told’ what to do rather than their ‘actual doing’: ‘*they just tell us what to do’* or *I’m not not allowed to have keys to the house, It’s the policy’*. Negative aligning cycles are indicative and counterbalanced by adults with lower levels of emotional stability adjusting less well in compliance situations. A negative aligning cycle maximizes the significance that compliance is a requirement of ‘being told’. These adults have fewer opportunities for self-direction, more experiences of external control of their choices, and are less influential in choice situations. The movement of the balance which exists, between framing control, emotional connecting and adjusting in compliance situations is therefore conditional upon the flexibility, rigidity and integrity of the boundaries of ‘being told’ and ‘actual doing’. The following presents an explanation of positive aligning cycles (Maria and Elaine) and negative aligning cycles (Saoirse and Sarah) punctuated with participant narratives and excerpts from interviews. Narratives and excerpts from interviews are also provided explaining how participants move from one cycle to another

### Positive aligning cycles

#### Embracing the flow of control

The first of two positive aligning cycles is presented in the

Embracing the flow of control denotes adult in control of choices made, are more emotionally stable and adjust very well in compliance situations. Their ‘actual doing’ is amplified as they consistently meet the requirements of ‘being told’ without needing to be told what to do. The following presents an example of embracing the flow of control.

Maria who is married and lives in an apartment in a retirement complex in the community. Maria has spent most of her life in residential setting but now lives independently and while she keeps links with a Registered Nurse (Intellectual Disability) she makes her own choices.

M I go shopping for my own clothes. I like going shopping on my own. I like shopping, I like shopping for bags and shoes. I love clothes. I like good quality clothes so I go to Dunne’s and to Penney’s. I prefer to go shopping on my own, it’s independence. I know what suits me and what doesn’t suit me and if you want help you ask the shop assistant.

In the above scenario Maria draws from a bank of ideas where her adult identity, her independent mindset has positive connotations. Along with the positivity of these frames of reference, she interprets the choice situation by targeting set practices and controls when, where and how she goes clothes shopping. The following explains how Maria uses rules to make choices:

M Eating healthy is very important. It’s good for the mind. I’ll tell you what I eat, in the morning I eat orange, an apple, a banana, and a diet yoghurt, for my tea break I’d have a kiwi or a mandarin orange, … … ., and you know those yoghurts you can drink to boost your cholesterol, yeah I like those, they are very good for you … . I cook fresh vegetables every day, I don’t like buying my vegetables in a package in a frozen compartment they are not good for you.

The following demonstrates emotional stability:

M It’s up to yourself to control your stress … … … … . When you get upset about something, you sit down and talk about it, go for a walk, play nice music, go for a bath, light a candle, What-ever plays on your mind don’t hide it … . you can’t keep it in, that will make it worse.

And adjusts in compliance situations:

M We sit down and we see what our finances are because all that comes into it. You can’t book anything you can’t afford and that’s very important … . We sit down and go through what money we have and where we want to go … … … … … . … … … You go through what money you have … .,

When what she does consistently succeeds in achieving and maintaining the requirements of what she is being told to do, staff telling her what to do (agency enacting practices) agency enacting practices are minimal and there is no obvious agency requirement of her to comply. The equilibrium moves towards being self-directed, as what she does has more significance, she is doing rather than being told to do it. As she is self-directed in what she does the equilibrium is maintained by a positive aligning cycle. She is totally confident and aware of her independence which positively feeds back into framing control, emotional stabilizing and model complying, which in turn, facilitates her sense of independence.

### Going with the flow of control

The second of two positive aligning cycles is presented in Figure 5

Going with the flow of control denotes adult engaging in self-control of choices made, are self-protecting and are functional in adjusting in compliance situations. Their ‘actual doing’ is amplified as they consistently begin to meet the requirements of what they are being told to do and continually informing agency in power positions that they are doing it.

The following presents an example of Elaine: ‘going with the flow of control’:

E I just ring staff and let them know where I am going and what time I will be back at. Staff tell me I can come and go as I please. The only thing is I lets staff know, it’s just for safety, I let them know where I’m gone and they don’t mind then you see, you know so … .

In the above scenario Elaine draws from a bank of ideas where her adult identity, her pragmatic mindset has positive connotations. Along with the positivity of these frames of reference she interprets choice situations by tracking transparency and engaging in self-control of the choice of going to meet her boyfriend. Elaine has a clear understanding of the unwritten rules and engages in reporting her whereabouts continually to staff. Elaine lives with two other adults in a community residential house, where staff stay just one night a week. Staff telling her what to do (agency enacting practices) is less intensified as there is less agency requirement of her to comply with what she is to do. The equilibrium moves towards her ‘actual doing’, as she functions within the remit that she is telling those in power positions what she is doing, rather than being told what to do. Elaine engaging in self-control is self-protective while adjusting well in compliance situations, she feels more secure that she lets staff know where she is going and what time she is back at. Elaine is cautiously gaining confidence and aware that she is gaining independence. This feeds back positively into framing control, emotional self-protecting and functional complying, which in turn, is facilitating opportunities for her to increase a sense of independence.

E I’d say down the road I’ll move in with him, I can see that happening all right. If I was getting married and moving in if I decide to stay with him I’d just say I’d like change and I’d like to stay with him.

### Negative aligning cycles

#### Getting carried by the flow of control

The first of two negative aligning cycles is presented in Figure 6

Getting carried by the flow of control’ denotes adults with beliefs of greater external control of choices they make, focus on their own self-preservation while they selectively adjust in compliance situations sometimes finding it difficult to do as told. ‘Being told’ is amplified as they are continually being told what to do, rather than actually doing it for themselves. The following presents an example of Saoirse: ‘getting carried by the flow of control’:

R And have you pain killers here with you today?

S No I’m not allowed to bring them to work … . It’s the policy … . if anybody took them or anything like that so I have to take them at home like … .

R And if you had a pain in your ear now what would you do

S I would have to wait … to ask for Panadol—I got two painkillers before I came to work this morning to be honest with you. The houseparent gave them to me this morning because I was complaining yesterday of a headache

R So you don’t manage your own medication in the house

S No, it’s the houseparent does that, she calls me to the office and I go and take them there

R And would you like to manage your own medication?

S Well if anyone took them I suppose by mistake, it’s the house policy, so nobody’s allowed to have tablets … in case somebody took them by mistake, it’s the rules, it’s only right, God forbid, like you know … if anybody take them by mistake and that’s probably why like.

It’s alright for the house-parents to have them, they can lock them away and take precautions like, its makes sense like, someone might take them when your not looking and then you might be blamed for something, cause that’s why the policy is there like … … … .

I hate to be complaining and looking for them but the doctor said to take them when I have the pain … when I like

The above explains how Saoirse draws from a bank of ideas where her adult identity, her dependent mindset has negative connotations. Saoirse interprets the choice situation with bounded understanding, as she determines there is greater external control regarding the management of her medication. She is of the understanding that the health board has implemented policies with rules to be abided by rendering her to be dependent on others for her medication management. Saoirse is accepting of the external control and externalizes control to those in positions of power, as she focuses on self-preservation feeling fearful of failure and feeling safer with the externalization of control to those in power positions. Along with the selective adjustments in compliance to specific requests, she submissively takes her medication when instructed. The following explains how Saoirse is sometimes challenged to meet or sustain what she is being told to do:

S I had the appointment today with a counsellor today and forgot about it

R What happened?

S XXX said: How dare you? Why weren’t you there? What did you go out for? You have to ring and apologize

R And what had happened that you had gone out?

S I had forgotten about it, I had forgotten about my appointment and XXXX came all the way out it was ridiculous. I had to ring to apologize.

When deviations are likely to occur between what Saoirse is being told and what she does, staff attitudes and practices intensify so as to bring Saoirse’s actual doing in line with what she is being told to do.

S Yea, it’s a bit much to be honest now I just feel that people are doing decisions for us. It’s like the mass like, like planning things ahead, … They had arranged mass without asking us in the community house, it’s at 6.30pm on Thursday, and a disco and buffet afterwards … . I wouldn’t lie. They didn’t ask us what did we want? Did we want to do it? And then they tell us ‘you have to come you can’t stay in the house on your own like’. It’s the rules with the health board and things are deciding for us. And XXX told me I was doing a reading, I said, I’m too nervous to do a reading in front of a crowd … . the words are too complicated … . with my ears I don’t like the noise … … XXX told me I have to do it. XXX said ‘YOU ARE DOING A READING’, the reading. I find some of the words are complicated and that’s putting me off. I’m only doing it to keep the peace.

The equilibrium continues to be maintained by a negative aligning cycle, wherein she is more guided by the fact that she is being told what to do rather than being self-directed in what she is doing. Whether satisfied or not with greater external control, such perceptions negatively feed back into framing control, counterbalanced by self-preservation and selective complying, and is limiting for Saoirse in achieving a sense of independence.

#### Resisting the flow of control

The second of two negative aligning cycles Figure7

Resisting the flow of control denotes adult responses when the reality of what they do is oppositional to what they are being told to do. The significant incongruence between what they do and what they are being told to do has consequences for these adults to believe that they are not in control of choice situations, are emotionally de-stabilizing, dissentious in adjustments they make in compliance situations. The equilibrium moves towards being told and lacking opportunities for self-direction, they either do as told or burst outside its boundaries engaging non-compliance. The following presents an example of Sarah ‘resisting the flow of control’:

S Would you stick that? I couldn’t stick that. I’m there only four months, I think, I don’t know when it started, I don’t know. I told XXXX like, to get me out and XXXX only made it a lot worse for me. I was only going for a day at a time, like a night here and a night there. I wasn’t meant to be temporary [means full time] at all like and XXXX got me put in full time. XXX said “do you want full time?” and I didn’t. (Sobbing) I was only supposed to be there part time and XXX made it full time. And XXXX was bringing her down on top of me and I didn’t want her at all … … . down on top of me—I don’t want her down on top of me I don’t. Look, I was supposed to be part-time, right, and I would go in there on a Friday and go home, no … . I used go in at the weekend, right, and go home on Monday right, and then XXXX do you know XXXX do you? And then XXXX made it worse I was only supposed to be there part time (Sobbing) for a night here and a night there like, to give my mother a break.

Sarah in the above scenario draws from a bank of ideas where her adult identity, oppressed mindset, and the lack of control she has in the choice situations have negative connotations. Along with the negativity of these frames of reference as Sarah interprets the choice situation she identifies powerlessness. Sarah recently moved to community residential services where she shares a bedroom with another lady. Sarah feels that she has been put into full-time community residential care against her will.

Sarah’s emotional distress is exacerbated by conflicting tensions regarding money management:

S … … . they have my money and they won’t give it to you when you ask them for it I mean do they? Like, they don’t like, like they give you f**k all, like my brother was in like and they don’t get it, and my mother was saying the same thing in like and they don’t get it and you see {researcher’s name} they have your money like and you don’t get it. And see if your looking for money you have to ask them … .they just don’t give it to you like. You have to ask for everything. It’s not good for me, it’s not good for me at all like.

Not in control, counterbalanced by emotional de-stabilizing is both indicative of and counterbalanced by dissentious complying, which ultimately feeds back into negatively framing control and the equilibrium is maintained at a negative aligning cycle.

R Do you have set meetings at all with your keyworker?

S They don’t do it. They don’t do it anymore

R And do you have a person centred plan or a lifestyle plan?

S No they don’t do it anymore. I tried that and it didn’t work.

At the outset, Sarah, presented as not being in control of choice situations. Extremely distressed with what was going on in her life, Sarah continually revisited these experiences of lacking choice and control. Dissentious in the adjustments she had to make to do as told Sarah maintained the equilibrium at a negative aligning cycle. This in turn is limiting for her in achieving a sense of independence. S: XXX asked me would I do art therapy? That’s a waste of money. I told him no, that wouldn’t work for me.

At a subsequent meetings Sarah informed the researcher that she had been put on medication for an underactive thyroid; introduced to a volunteer friend whom she enjoyed meeting; made friends with her roommate whose mother had brought her a present. She still wanted to ‘*get out’* of community residential services and have ‘*my independence’*. Sarah reported that her keyworker had with her, signed a letter for higher-level management within the service requesting her wishes to ‘*get out’* of the community residential services.

## Discussion

The grounded theory of ‘aligning with the flow of control’ accounts for the continuous negotiation of the adults’ main concern; that of control in environments that are controlling of them. Having differing levels of control in the context of different choice situations, it is important to note that they are never either entirely person-driven or entirely other driven, but are constantly engaged in negotiation between the two. Aligning with the flow of control is not a fixed state, but is conceptualized as a dynamic equilibrium.

As an internal control psychology, Choice Theory (Glasser, 1998) has been developed as a theory to replace external control psychology, while demonstrating how choice and control are inextricably linked. Originally Dr. William Glasser an American Psychiatrist developed Reality Therapy as a counselling mechanism while working with patients diagnosed with mental illness and subsequently founded the Institute for Reality Therapy. Reality therapy is a method of counselling which teaches people how to direct their own lives, make more effective choices, and how to develop the strength to handle the stresses and problems of life. As his theoretical thinking around the underpinnings of Reality Therapy developed Dr Glasser based its’ philosophical underpinnings on the theory named Choice Theory and changed the name of the Institute from Reality Therapy to the Choice Theory Institute (Glasser, 1998). Similarly, this emergent theory characterizes decision-making practices of adults with intellectual disabilities as those who create or maintain an equilibrium based on their control beliefs, emotions and the adjustments they make in compliance situations while accessing services and support.

This theory illustrates a spectrum of autonomy explicating complexities and intricacies of decision-making support as reported by individuals with varying needs, abilities and support networks. Research has been undertaken on measuring choice availed by people with intellectual disabilities (Houseworth et al., 2018) and understanding components of choice-making (Agran et al., 2010; Cobigo et al., 2010; Donelly et al., 2010; McCarthy, 2010; McConkey & Collins, 2010; Wiltz & Kalnins, 2008). Paradigm shifts in service provision such as personalization (Williams & Porter, 2017), self-directed support (Bogenschutz et al., 2019) and supported decision-making (Bigby et al., 2019; Carey & Ryan, 2019; Curryer et al., 2019; Gore, 2008; Shogren et al., 2017; Watson, 2016) provide guidance so that legal and valued entitlements such as autonomy are implemented with and for service users. Greater focus is now on the recognition and development of interactions in the promotion of self-determination (Cudré‐Mauroux et al., 2019). This theory provides for greater understanding of choice and control from the perspectives of people with intellectual disabilities, therefore, service providers may better create and sustain enabling conditions so that people with intellectual disability are facilitated to establish trusting relationships and experience personal autonomy.

This emergent theory of ‘aligning with the flow of control’ advocates that in order to have the universality of human rights realized for people with intellectual disabilities the concepts of choice and control from a lens perspective of facilitating autonomy must be progressed. The findings reported in this paper are of direct relevance for people with intellectual disabilities and their support network, particularly in Ireland in the context of the roll out of the Assisted Decision Making (Capacity) Act (Department of Justice, 2015). As new self-directed support arrangements will be rolled-out over time a nuanced and flexible understanding is essential if service providers and those supporting decision-making with adults with intellectual disabilities are to maximize opportunities for exercising choice and control, while ensuring safeguarding are in place (Bach & Kerzner, 2010; Flynn & Arstein-Kerslake, 2014a).

Glasser (1998) believes that when individual requirements of genetically driven needs are accommodated, internal control psychology is developed individuals find satisfaction in their relationships with relevant others. This theory found that a greater sense of internal control is indicative to having greater satisfaction in building trusting relationships is especially true for people with intellectual disabilities. Service providers need to understand the extent to which autonomy can impact on the establishment of trusting relationships, especially for those adults who are more vulnerable to exploitation. These findings offer a sort of counter-point to the notion of “supported decision-making” a now prominent element of policy and decision-making literature relevant to adults with intellectual disabilities (Bach & Kerzner, 2010; Carey & Ryan, 2019; Flynn & Arstein-Kerslake, 2014a, 2014b). Developing structured forums within supportive services where matters of choice and control can be creatively discussed and debated aiming to enhance autonomy while ensuring safeguarding is vitally important.

While this research data was collected in 2012, presenting a theory which explains choice and autonomy in decision-making as experienced by a group of adults with intellectual disabilities who are in receipt of service provision provides support for Irelands continuing commitment to ensure the human rights of people with disabilities are enshrined in everyday practices (UNCRPD, 2006).

### Implications

Human dignity is now recognized internationally as the foundation upon which human rights are based capturing the notion that every human being is unique and valued (Roberto, 2014). By ensuring that people’s autonomy is respected, service providers continually improve the quality of care, safety and quality of life of adults who access services. Providers of services to adults with intellectual disabilities must ensure that the inalienable rights of these vulnerable service users are embedded in the culture of the organization, whether that be in the development of practice standards or the implementation and evaluation of quality assurance mechanisms. It is the authors belief that continuing focus on human rights will ensure that both service providers and service users become increasingly aware of their individual responsibilities in ensuring, promoting protecting and enabling people with intellectual disabilities to live lives which are equal to and experienced by other citizens in their communities.

## Conclusion

The theory presented within this article highlights the dilemmas, of adult research participants, accessing services, availing of supports, while having differing levels of control in the context of making life choices. These adults use frames of reference and make interpretations, and determine the level of control they have in choices situations. And with emotional connecting and adjusting in compliance the state of equilibrium is ultimately determined as they respond to what they are told to do and perceive differing levels of satisfaction and success with choice outcome. The complexity of human situations means that their choices are not always simple and straightforward. Service providers need to have structures and processes in place which aim to understand each persons’ beliefs of control relevant to each choice situation and to ensure time and space are made available to the person and to those who support them so as to facilitate a process of learning which in turn will enable the formation of trusting and satisfying relationships. When supporting people with intellectual disabilities to make choices, it is significantly important to understand how these adults cope emotionally with control beliefs and the impact of compromises and adjustments they make which iteratively feed back into how they frame control. Additionally, further research be undertaken to assist service providers to evaluate their services, demonstrate accountability, and to maintain a balance between promoting autonomy and safeguarding. More educational focus is required for adults with intellectual disabilities and those who support them to find ways to enable people be more self-directed in their own lives, to make meaningful choices which have impact and outcomes, and how to be enabled to develop skills to cope to enhance mental health and well-being while coping emotionally with stresses and problems of life.

Limitations of this study are the small sample size along with the dominance of women as research participants. Future studies should be undertaken to assess whether any significant differences exist between men and women and how they respond to matters of control in their life choices. It is also needs to be considered that the location of the study was undertaken in a society with active measures to enhance the autonomy of adults with intellectual disability the situation might be quite different in less supportive environments where varying policies may have an impact. It is paramount that research undertaken on the role of family members and social supports impacting these cycles.

## References

[cit0001] Agran, M., Storey, K., & Krupp, M. (2010). Choosing and choice making are not the same: Asking “what do you want for lunch?” is not self-determination. *Journal of Vocational Rehabilitation*, 33(2), 77–17. 10.3233/JVR-2010-0517

[cit0002] Alonso-Sardón, M., Iglesias-de-Sena, H., Fernández-Martíet, L. C., & Mirón-Canelo, J. A. (2019). Do health and social support and personal autonomy have an influence on the health related quality of life of individuals with intellectual disability? *BMC Health Services Research*, 19(63), 1–10. 10.1186/s12913-018-3856-5 30674320 PMC6345008

[cit0003] Antaki, C., Finlay, W. M. L., & Walton, C. (2009). Choices for people with intellectual disabilities: Official discourse and everyday practice. *Journal of Policy and Practice in Intellectual Disabilities*, 6(4), 260–266. 10.1111/j.1741-1130.2009.00230.x

[cit0004] Bach, M., & Kerzner, L. (2010). *A new paradigm for protecting autonomy and the right to legal capacity prepared for the law commission of Ontario*. The Ontario Law Commission, Canada. Retrieved 46, 2018, from http://www.1co-cdo.org/en/disabilities-call-for-papers-bach-kerzner

[cit0005] Bigby, C., Whiteside, M., & Douglas, J. (2019). Providing support for decision making to adults with intellectual disability: Perspectives of family members and workers in disability support services. *Journal of Intellectual & Developmental Disability*, 44(4), 396–409. 10.3109/13668250.2017.1378873

[cit0006] Bjornsdottir, K., Stefansdottir, G., & Stefansdottir, A. (2015). It’s my life’: Autonomy and people with intellectual disabilities. *Journal of Intellectual Disabilities*, 19(1), 5–21. 10.1177/1744629514564691 25542701

[cit0007] Bogenschutz, M. D., DeCarlo, M., Hall-Lande, J., & Hewitt, A. (2019). Fiscal stewardship, choice, and control: The context of self-directed services for people with intellectual and developmental disabilities in the USA. *Intellectual and Developmental Disabilities*, 57(2), 158–171. 10.1352/1934-9556-57.2.158 30920908

[cit0008] Brown, I., & Brown, R. (2009). Choice as an aspect of quality of life for people with intellectual disabilities. *Journal of Policy and Practice in Intellectual Disability*, 6(1), 11–18. 10.1111/j.1741-1130.2008.00198.x

[cit0009] Carey, E. (2010). Navigating the process of ethical approval: A methodological note. *The Grounded Theory Review*, 9(3), 19–32.

[cit0010] Carey, E., & Ryan, R. (2019). Chapter 14 informed consent. In J. L. Matson (Ed.), *Handbook of intellectual disabilities integrating theory, research and practice*. Springer. 221–247

[cit0011] Carey, E., & Griffiths, C. (2016). The impact of Irish policy and legislation on how adults with learning disabilities make choices. *British Journal of Learning Disabilities*, 44(2), 111–121. 10.1111/bld.12117

[cit0012] Carey, E., & Griffiths, C. (2017). Recruitment and consent of adults with intellectual disabilities in a classic grounded theory research study: Ethical and methodological considerations. *Disability & Society*, 32(2), 193–212. 10.1080/09687599.2017.1281793

[cit0013] Carney, T. (2013). Participation and service access rights for people with intellectual disability: A role for law? *Journal of Intellectual & Developmental Disability*, 38(1), 59–69. 10.3109/13668250.2012.738810 23164093

[cit0014] Chapman, M., Bannister, S., Davies, J., Fleming, S., Graham, C., McMaster, A., Seddon, A., Wheldon, A., & Whittell, B. (2011). Speaking up about advocacy: Findings from a partnership research project. *British Journal of Learning Disabilities*, 40(1), 71–78. 10.1111/j.1468-3156.2011.00688.x

[cit0015] Chou, U.-C., Pu, C., Kroger, T., Lee, W., & Chang, S. (2011). Outcomes of a new residential scheme for adults with intellectual disabilities in Taiwan: A 2 year follow-up. *Journal of Intellectual Disability Research*, 55(9), 823–831. 10.1111/j.1365-2788.2011.01394.x 21366754

[cit0016] Chou, Y. C., & Lu, Z. Y.. (2011). Deciding about sterilisation: Perspectives from women with an intellectual disability and their families in Taiwan. *Journal of Intellectual Disability Research*, 55(1), 69–74. 10.1111/j.1365-2788.2010.01347.x 21121994

[cit0017] Ciulla, J., Timmons, A., Hall, C., Bose, J., Wolfe, A., & Winsor, J. (2011). Choosing employment: Factors that impact employment decisions for individuals with intellectual disabilities. *Intellectual and Developmental Disabilities*, 49(4), 285–299. 10.1352/1934-9556-49.4.285 21721981

[cit0018] Cobigo, V., Lachapelle, Y., & Morin, D. (2010). Choice-making in vocational activities planning: Recommendations from job coaches. *Journal of Policy and Practice in Intellectual Disabilities*, 27(4), 245–249. 10.1111/j.1741-1130.2010.00273.x

[cit0019] Conder, J., Mirfin-Veitch, B., Sanders, J., & Munford, R. (2010). Planned pregnancy, planned parenting; enabling choice for adults with a learning disability. *British Journal of Learning Disabilities*, 39(2), 105–112. 10.1111/j.1468-3156.2010.00625.x

[cit0020] Cudré‐Mauroux, A., Piérart, G., & Vaucher, C. (2019). The importance of the relational needs of people with learning disabilities in the promotion of self‐determination. *British Journal of Learning Disabilities*, 47(3), 174–180. 10.1111/bld.12268

[cit0021] Curryer, B., Stancliffe, R. J., Dew, A., & Wiese, M. (2019). Self-determination of adults with intellectual disability: Impact of family relationships. *Journal Of Intellectual Disability Research*, 63(7), 746–747. DOI:10.1111/jir.12657.

[cit0022] Department of Justice. (2015). Assisted decision making (Capacity) act (ADMA). The Stationery Office. Retrieved 311, 2020, from http://www.oireachtas.ie/viewdoc.asp?DocID=30677&ad=1

[cit0023] Donelly, M., Hillman, A., Stancliffe, R. J., Knox, M., Whitaker, L., & Parmenter, T. R. (2010). The role of informal networks in providing effective work opportunities for people with an intellectual disability. *Work*, 36(2), 227–237. 10.3233/WOR-2010-1023 20634616

[cit0024] Feldman, M. A., Owen, F., Andrews, A., Hamelin, J., Barber, R., & Griffiths, D. (2012). Health self-advocacy training for persons with intellectual disabilities. *Journal of Intellectual Disability Research*, 56(11), 1110–1121. 10.1111/j.1365-2788.2012.01626.x 23106754

[cit0025] Flynn, E., & Arstein-Kerslake, A. (2014a). Legislating personhood: Realising the right to support in exercising legal capacity. *International Journal of Law in Context*, 10(1), 81–104. 10.1017/S1744552313000384

[cit0026] Flynn, E., & Arstein-Kerslake, A. (2014b). The support model of legal capacity: Fact, fiction, or fantasy? *Berkeley Journal of International Law*, 32, 134–281.

[cit0027] Frielink, N., Schuengel, C., & Embregts, P. J. C. M. (2017). Autonomy support in people with mild-to-borderline intellectual disability: Testing the health care climate questionnaire-intellectual disability. *Journal of Applied Research in Intellectual Disabilities : JARID*, 2018(31), 159–163. DOI:10.1111/jar.12371 28544437

[cit0028] Frielink, N., Schuengel, C., & Embregts, P. J. C. M. (2018). Autonomy support, need satisfaction, and motivation for support among adults with intellectual disability testing a self-determination theory model. *American Journal on Intellectual and Develomental Disabilities*, 123(1), 33–49. 10.1352/1944-7558-123.1.33 29281319

[cit0029] Glaser, B. G. (1978). *Theoretical sensitivity: Advances in the methodology of grounded theory*. Sociology Press.

[cit0030] Glaser, B. G. (1998). *Doing grounded theory: Issues and discussions*. Sociology Press.

[cit0031] Glaser, B. G. (2001). *The grounded theory perspective: Conceptualization contrasted with description*. Sociology Press.

[cit0032] Glaser, B.G. (1967). *The discovery of grounded theory: Strategies for qualitative research*. Hawthorne, New York: Aldine de Gruyther

[cit0033] Glasser, W. (1998). *Choice theory a new psychology of personal freedom*. Harper Collins.

[cit0034] Gore, N. (2008). Financial decision‐making: Guidance for supporting financial decision‐making by people with learning disabilities. *Journal of Intellectual Disability Research*, 52(3), 273–274. 10.1111/j.1365-2788.2007.01029.x

[cit0035] Gumbis, J., Bacianskaite, V., & Randakeviciute, J. (2017). Do human rights guarantee autonomy? *Vilnius*, 62/63, 77–93.

[cit0036] Hatton, C., Emerson, E., Robertson, J., Gregory, N., & Kessissoglou, S. (2004). The resident choice scale: A measure to assess opportunities for self-determination in residential settings. *Journal of Intellectual Disability Research*, 48(2), 103–113. 10.1111/j.1365-2788.2004.00499.x 14723653

[cit0037] Hawkins, R., Redley, M., & Holland, A. J. (2011). Duty of care and autonomy: How support workers managed the tension between protecting service users from risk and promoting their independence in a specialist group home. *Journal of Intellectual Disability Research*, 55(9), 873–884. 10.1111/j.1365-2788.2011.01445.x 21726324

[cit0038] Health Information and Quality Authority (HIQA). (2019). ‘Guidance on a human rights based approach in health and social care services (HIQA 2019). Health Information and Quality Authority. Retrieved 311, 2020, from https://www.hiqa.ie/sites/default/files/2019-11/Human-Rights-Based-Approach-Guide.PDF

[cit0039] Health Service Executive (HSE). (2018). Transforming lives programme to implement the recommendations of the value for money and policy review of disability services in Ireland. Health Service Executive. Retrieved 327, 2020, from https://www.hse.ie/eng/services/publications/effective-participation-in-decision-making-final.pdf

[cit0040] Houseworth, J., Stancliffe, R. J., & Ticháa, R. (2018). Association of state-level and individual-level factors with choice making of individuals with intellectual and developmental disabilities. *Research in Developmental Disabilities*, 83, 77–90. 10.1016/j.ridd.2018.08.008 30144747

[cit0041] Irish Human Rights Equality Commission (IHREC). (2019). Implementing the public sector equality and human rights duty. Retrieved 311, 2020, from. https://www.ihrec.ie/app/uploads/2019/03/IHREC_Public_Sector_Duty_Final_Eng_WEB.pdf

[cit0042] Jenkinson, J. (1993). Who shall decide? The relevance of theory and research to decision-making by people with an intellectual disability. *Disability, Handicap & Society*, 8(4), 361–375. 10.1080/02674649366780351

[cit0043] Jenkinson, J., Copeland, C., Drivas, V., Scoon, H., & Yap, M. L. (1992). Decision-making by community residents with an intellectual disability. *Australia and New Zealand Journal of Developmental Disabilities*, 18(1), 1–8. 10.1080/07263869200034761

[cit0044] Jenkinson, J., & Nelms, R. (1994). Patterns of decision-making behaviour by people with intellectual disability: An exploratory study. *Australia and New Zealand Journal of Developmental Disabilities*, 19(2), 99–109. 10.1080/07263869400035141

[cit0045] Jensen, C. C., Lydersen, T., Johnson, P. R., Weiss, S. R., Marconi, M. R., Cleave, M. L., & Weber, P. (2012). Choosing staff members reduces time in mechanical restraint due to self-injurious behaviour and requesting restraint. *Journal of Applied Research in Intellectual Disabilities*, 25(3), 282–287. 10.1111/j.1468-3148.2011.00664.x 22489039

[cit0046] Johnson, K. R., & Bagatell, M. (2020). No! You can’t have it”: Problematizing choice in institutionalized adults with intellectual disabilities. *Journal of Intellectual Disabilities*, 24(1), 69–84. 10.1177/1744629518766121 29621910

[cit0047] Kilcommons, A. M., Withers, P., & Moreno-Lopez, A. (2012). Do service users with intellectual disabilities want to be involved in the risk management process? A thematic analysis’. *Journal of Applied Research in Intellectual Disabilities*, 25(5), 433–444. 10.1111/j.1468-3148.2012.00684.x 22890944

[cit0048] Kirkendall, A., Linton, K., & Farris, S. (2017). Intellectual disabilities and decision making at end of life: A literature review. *Journal of Applied Research in Intellectual Disabilities*, 30(6), 982–994. 10.1111/jar.12270 27456315

[cit0049] Lysaght, R., Ouellette-Kuntz, H., & Morrison, C. (2009). Meaning and value of productivity to adults with intellectual disabilities. *Intellectual and Developmental Disabilities*, 47(6), 413–424. 10.1352/1934-9556-47.6.413 20020797

[cit0050] McCarthy, M. (2010). Exercising choice and control –women with learning disabilities and contraception. *British Journal of Learning Disabilities*, 38(4), 293–302. 10.1111/j.1468-3156.2009.00605.x

[cit0051] McConkey, R., & Collins, S. (2010). Using personal goal setting to promote the social inclusion of people with intellectual disabilities living in supported accommodation. *Journal of Intellectual Disability Research*, 54(2), 135–143. 10.1111/j.1365-2788.2009.01224.x 19874448

[cit0052] McDaniels, B. (2016). Disproportionate opportunities: Fostering vocational choice for individuals with intellectual disabilities. *Journal of Vocational Rehabilitation*, 45(1), 19–25. 10.3233/JVR-160807

[cit0053] Michell, B. (2012). Checking up on des: My life my choice’s research into annual health checks for people with learning disabilities in Oxfordshire. *British Journal of Learning Disabilities*, 40, 152–161. 10.1111/j.1468-3156.2012.00742.x

[cit0054] Moore, K. L. (2019). Disabled autonomy. *Journal of Health Care Law & Policy*, *22*(2), 245–279. heinonline.org/HOL/Page?handle=hein.journals/hclwpo22&div=26&g_sent=1&casa_token=&collection=journals

[cit0055] Nursing and Midwifery Board of Ireland (NMBI). (2014). *Code of professional conduct and ethics for registered nurses and registered midwives*. Nursing and Midwifery Board of Ireland.

[cit0056] O’Donovan, M. A., Byrne, E., McCallion, P., & McCarron, M. (2017). Measuring choice for adults with an intellectual disability –a factor analysis of the adapted daily choice inventory scale. *Journal of Intellectual Disability Research*, 61(5), 471–487. 10.1111/jir.12364 28281320

[cit0057] Owen, K., Hubert, J., & Hollins, S. (2008). Moving home: The experiences of women with severe intellectual disabilities in transition from a locked ward. *British Journal of Learning Disabilities*, 36(4), 220–226. 10.1111/j.1468-3156.2007.00484.x

[cit0058] Redley, M., Maina, E., Keeling, A., & Pattni, P. (2012). The voting rights of adults with intellectual disabilities: Reflections on the arguments, and situation in Kenya and England and Wales. *Journal of Intellectual Disability Research*, 56(11), 1026–1035. 10.1111/j.1365-2788.2012.01635.x 23106747

[cit0059] Roberto, A. (2014). Human dignity and human rights. In H. A. M. J. Ten Have & B. Gordijn (Eds.), *Handbook of global bioethics* (pp. 45–57). Springer.

[cit0060] Rogers, E., Pilch, M., McGuire, B. E., Flynn, E., & Egan, J. (2020). Psychologists‘ perspectives on supported decision making in Ireland. *Journal of Intellectual Disability Research*, 64(3), 234–245. 10.1111/jir.12712 31975473

[cit0061] Shogren, K., Wehmeyer, M., Lassmann, H., & Forber-Pratt, A. (2017). Supported decision making: A synthesis of the literature across intellectual disability, mental health, and aging. *Education and Training in Autism and Developmental Disabilities*, 52(2), 144–157. jstor.org/stable/26420386?seq=1#metadata_info_tab_contents

[cit0062] Stancliffe, R. J., Lakin, K. C., Larson, S., Engler, J., Taub, S., & Fortune, J. (2011). Choice of living arrangements. *Journal of Intellectual Disability Research*, 55(8), 746–762. 10.1111/j.1365-2788.2010.01336.x 21029234

[cit0063] Suto, W. M. I., Clare, I. C. H., Holland, A. J., & Watson, P. C. (2005). The relationships among three factors affecting the financial decision‐making abilities of adults with mild intellectual disabilities. *Journal of Intellectual Disability Research*, 49(3), 210–217. 10.1111/j.1365-2788.2005.00647.x 15713196

[cit0064] United Nations Convention on the Rights of Persons with Disabilities (UNCRPD). (2006). United Nations. Retrieved 311, 2020, from www.un.org/disabilities/convention/conventionfull.shtml

[cit0065] Watson, J. (2016). Assumptions of decision-making capacity: The role supporter attitudes play in the realisation of article 12 for people with severe or profound intellectual disability. *Laws*, 5(1), 6. 10.3390/laws5010006

[cit0066] Webber, C., & Cobigo, V. (2014). What should service providers know when measuring how they impact consumers’ freedom to make choices? *Journal on Developmental Disabilities*, 20(2), 8–19.

[cit0067] Whitehead, L. C., Trip, H. T., Hale, L. A., & Conder, J. (2016). Negotiated autonomy in diabetes self‐management: The experiences of adults with intellectual disability and their support workers. *Journal of Intellectual Disability Research*, 60(4), 389–397. 10.1111/jir.12257 26840793

[cit0068] Williams, V., & Porter, S. (2017). The meaning of ‘choice and control’ for people with intellectual disabilities who are planning their social care and support. *Journal of Applied Research in Intellectual Disabilities*, 30(1), 97–108. 10.1111/jar.12222 26500151

[cit0069] Wiltz, J., & Kalnins, T. (2008). Crisis management aggression, sociability, and roommate friendship: New findings translated into a resource for self-determined choices. *Journal of Policy and Practice in Intellectual Disabilities*, 5(3), 159–166. 10.1111/j.1741-1130.2008.00168.x

[cit0070] Zolkefli, Y. (2017). Evaluating the concept of choice in healthcare. *The Malaysian Journal of Medical Sciences : MJMS*, 24(6), 92–96. 10.21315/mjms2017.24.6.11 PMC5771520 29379391

